# Efficacy and safety of Iguratimod in the treatment of Ankylosing Spondylitis: A systematic review and meta-analysis of randomized controlled trials

**DOI:** 10.3389/fimmu.2023.993860

**Published:** 2023-03-03

**Authors:** Zhiyong Long, Ying Deng, Qi He, Kailin Yang, Liuting Zeng, Wensa Hao, Yuxuan Deng, Jiapeng Fan, Hua Chen

**Affiliations:** ^1^ Department of Rehabilitation Medicine, Guangzhou Panyu Central Hospital, Guangzhou, China; ^2^ People's Hospital of Ningxiang City, Ningxiang, China; ^3^ Hunan University of Chinese Medicine, Changsha, China; ^4^ Department of Rheumatology and Clinical Immunology, Peking Union Medical College Hospital, Chinese Academy of Medical Sciences & Peking Union Medical College, Beijing, China; ^5^ Qiqihar Medical University, Qiqihar, China; ^6^ ZCCC Jinzhu Transportation Construction Co. Ltd., Hangzhou, Zhejiang, China

**Keywords:** Iguratimod, ankylosing spondylitis, systematic review, meta-analysis, randomized controlled trial

## Abstract

**Objective:**

To explore the efficacy and safety of Iguratimod (IGU) intervention in the treatment of Ankylosing Spondylitis (AS).

**Methods:**

We used computer to search literature databases, collected randomized controlled trials (RCTs) related to IGU treatment of AS, and searched the relevant literature in each database until Sep. 2022. Two researchers independently carried out literature screening, data extraction, and evaluation and analysis of the risk of bias in the included studies, and then used Rev Man5.3 software for meta-analysis. The protocol is CRD42020220798.

**Results:**

A total of 10 RCTs involves in 622 patients were collected. The statistical analysis showed that IGU can decrease the BASDAI score (SMD -1.62 [-2.20, -1.05], P<0.00001. Quality of evidence: low), the BASFI score (WMD -1.30 [-1.48, -1.12], P<0.00001. Quality of evidence: low) and the VAS (WMD -2.01 [-2.83, -1.19], P<0.00001. Quality of evidence: very low). Meanwhile, the addition of IGU into the conventional therapy would not increase the adverse events (RR 0.65 [0.43, 0.98], P=0.04. Quality of evidence: moderate).

**Conclusion:**

IGU may be an effective and safe intervention for AS.

**Systematic review registration:**

https://www.crd.york.ac.uk/prospero/display_record.php?, identifier CRD42020220798.

## 1 Introduction

Ankylosing Spondylitis (AS) is a chronic inflammatory autoimmune disease, which mainly involves axial joint involvement, which may be accompanied by extra-articular manifestations. In severe cases, spinal deformity and joint stiffness may occur. One of the current features of AS is the high prevalence rate (0.86% in Western European white population) and the low incidence rate (1, 2). Patients can live with the disease for many years, and fusion of the spine or peripheral joints can occur in the late stage, causing the patient to lose motor function and living ability, and bring a heavy economic burden to the family and society (3, 4). Inflammation and pathological new bone formation are the two most important pathological features of AS. The early stage of AS is mainly manifested by inflammation and the bone erosion and destruction caused by it, and the late stage causes ectopic new bone formation (4, 5). As the initiating factor, inflammation runs through the entire process of disease development. There are many studies on it at present. The research on pathological new bone formation and the development of corresponding therapeutic drugs are still in the initial stage (5).

The therapeutic drugs for AS currently used clinically mainly include non-steroidal anti-inflammatory drugs (NSAIDs) and biological agents (TNF-α blockers). Although these drugs have achieved good anti-inflammatory effects, they have certain limitations and side effects, and there is no clear evidence for the role of AS new bone formation (6, 7). NSAIDs, as the first-line drugs recommended by AS treatment guidelines, have good anti-inflammatory and analgesic effects, but they need to be taken for a long time and have side effects such as cardiovascular, gastrointestinal and renal toxicity (8, 9). Similarly, TNF-α blockers are not effective for some patients, they are also very expensive, and there are reports that they may increase the risk of cancer (10). Therefore, new drugs for the treatment of AS are urgently needed clinically.

Iguratimod (IGU) is a new type of small molecule anti-rheumatic drug, which has the effects of non-steroidal anti-inflammatory drugs (NSAID) and disease mitigating anti-rheumatic drugs (DMARD). At present, it has been widely used clinically in China and Japan for the treatment of rheumatoid arthritis (RA) (11). IGU not only inhibits related inflammation-related signaling pathways and the expression of inflammatory factors (NF-κB and IL-17 inflammatory signaling pathways) (12), but also inhibits osteoclast differentiation (RANKL signaling pathway), promote osteoblast function (BMP/Dlx5/Osterix signaling pathway), and reduce cartilage destruction (MMPs family related factors) (13, 14), so as to play a bone protection role. At present, clinical randomized controlled trial (RCT)s showed the efficacy of IGU on AS (15–24), but there is no relevant research to systematic review and meta-analyze these RCTs to provide new evidence. Therefore, this research will evaluate the effectiveness and safety of IGU intervention in AS through systematic reviews and meta-analysis for the first time, in order to provide new evidence for clinical use.

## 2 Materials and methods

### 2.1 Protocol

This systematic review and meta-analysis were conducted strictly in accordance with the protocol [CRD42020220798 (see **Supplementary Material**)].

### 2.2 Search criteria

(1) Participants: Patients diagnosed with AS. All patients are at least 18 years old, and there are no restrictions on gender, race, and region. (2) Intervention methods: The intervention of the experimental group is IGU, used alone or in combination with the control group’s drugs. The intervention of the control group was conventional therapy. (3) Outcomes: Bath Ankylosing Spondylitis Disease Activity Index (BASDAI), Bath Ankylosing Spondylitis Functional Index (BASFI), VAS and adverse events; secondary outcomes are ESR, CRP, TNF-α, back pain score, SOD, CTX-I, β⁃CTX, OPG. (4) Study design: Randomized controlled trial without any limitations.

### 2.3 Literature search and screening strategy

We searched the ClinicalTrials, the China National Knowledge Infrastructure Databases (CNKI), Web of Science, Pubmed, The Chinese Science and Technology Periodical Database (VIP), EMBASE, Wan Fang Database, CiNii Research, J-STAGE, National Diet Library Digital Collections (NDLDC), Chinese Biomedical Database (CBM), Medline Complete, Cochrane Library. The retrieval time is up to Sep. 2022. The search strategy was shown in **Table S1**. All included studies were screened by two researchers according to the search criteria. If there is a disagreement between the two, the two researchers will discuss and resolve with the other researchers.

### 2.4 Data extraction and quality assessment

In order to collect the sample size, baseline conditions, treatment plan, treatment time, outcomes and other information included in the RCTs, a table was made to facilitate the extraction of relevant data and retrieval records. Data extraction was carried out independently by two researchers, and differences were resolved through discussions with other researchers. RCT quality assessment is carried out according to the risk of bias tool included in the Cochrane Handbook or Systematic Reviews of Interventions Version (25). The following aspects are evaluated for each study: random sequence generation and allocation hiding (selection bias), blinding (performance bias and detection bias), incomplete outcome data (detection bias), selective reporting (reporting bias) and other bias. The results of the analysis are divided into: “yes” (low risk of bias), “no” (high risk of bias), and “unclear” (unknown risk of bias).

### 2.5 Statistical analysis

Review Manager 5.3 software was used for statistical analysis (26). For continuous variables such as BASDAI, BASFI, VAS, ESR, CRP, the mean difference (MD) was used to describe the effect size, and the confidence interval (CI) is 95%. For dichotomous variables such as adverse event indicators, relative risk (RR) was used to describe the impact, and the CI is set to 95%. The χ^2^ test was used to analyze the heterogeneity between the results. In the case of low heterogeneity (P>0.1, I2<50%), a fixed effects model analysis was performed. If there is heterogeneity between the studies, a random effects model was used. The publication bias was detected by STATA 15 with Egger method (continuous variable) and Harbord methods (dichotomous variable) for primary outcomes. P>0.1 is considered to have no publication bias.

## 3 Results

### 3.1 Results of the search and description of included trials

**Figure 1:**
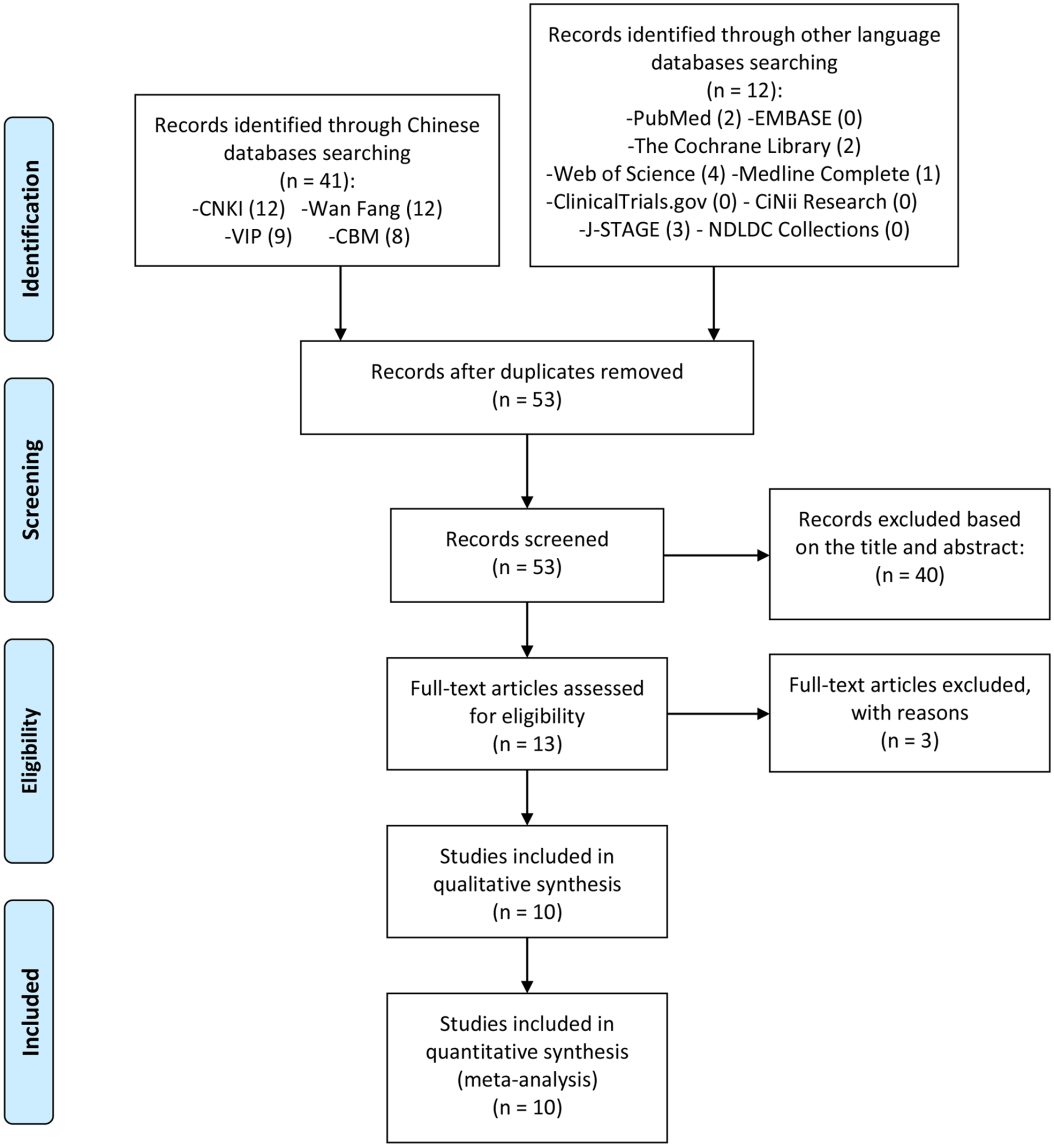
Flow diagram.

The total records identified through database searching and other sources were 53. Forty (40) were excluded based on the title and abstract and 13 for more detailed evaluation. Three (3) of 13 records were excluded because they were not RCTs (27–29) (**Figure 1**). All patients in those RCTs come from China and involves in 622 participants. The age range of patients is 20-50 years old, and the course of treatment is at least 12 weeks and the maximum is 24 weeks. The details of study characteristics are presented in **Table 1**.

**Table 1:** The characteristics of the included studies.

Study	Country	Sample size		Intervention		Relevant outcomes	Mean age (years)		BASDAI		BASFI		Duration
		Trial group	Control group	Trial group	Control group		Trial group	Control group	Trial group	Control group	Trial group	Control group	
Qiu et al., 2016 (15)	China	18	18	Iguratimod 25mg B.i.d	NSAIDs+DMARDs	ESR, BASDAI, BASFI, VAS, back pain score, adverse events	37.3 ± 7.0	34.5 ± 9.3	5.4 ± 1.1	5.6 ± 1.1	4.9 ± 1.9	5.0 ± 1.7	24 weeks
Yuan et al., 2020 (16)	China	41	39	Iguratimod 25mg B.i.d + Etoricoxib tablets 60 mg Q.d. + ibuprofen 300 mg T.i.d. + methotrexate 15 mg once a week	Etoricoxib tablets 60 mg Q.d. + ibuprofen 300 mg T.i.d. + methotrexate 15 mg once a week	VAS, CRP, ESR, SOD, CTX-I, adverse events	39.28 ± 5.30	40.08 ± 5.67	–	–	–	–	12 weeks
Pang et al., 2020 (17)	China	39	39	Iguratimod 25mg B.i.d + Etanercept 25mg tiwce a week	Etanercept 25mg tiwce a week	ESR, CRP, BASDAI, β-CTX, OPG, TNF⁃α	24.85 ± 4.18	25.01 ± 4.29	6.22 ± 1.38	6.19 ± 1.28	–	–	12 weeks
Lin et al., 2019 (18)	China	24	24	Iguratimod 25mg B.i.d + Sulfasalazine 1 g B.i.d. + methotrexate 10 mg once a week + NSAIDs	Sulfasalazine 1 g B.i.d. + methotrexate 10 mg once a week + NSAIDs	BASDAI, BASFI, VAS, adverse events	32. 71 ± 8. 80	28. 21 ± 6. 69	5. 25 ± 1. 03	5. 29 ± 1. 02	4. 85 ± 1. 56	4. 62 ± 1. 34	24 weeks
Xu et al., 2019 (19)	China	21	21	Iguratimod 25mg B.i.d + Celecoxib 0.2 g Q.d.	Sulfasalazine 1 g B.i.d. + Celecoxib 0.2 g Q.d.	BASDAI, BASFI, VAS, ESR, CRP, adverse events	35.1± 10.3	34.3± 9.5	5.5± 0.9	5.6± 0.9	5.9± 1.5	6.1± 1.3	24 weeks
Zeng et al., 2016 (20)	China	25	25	Iguratimod 25mg B.i.d + Meloxicam 7.5 mg Q.d.	Sulfasalazine 0.75 g T.i.d. + Meloxicam 7.5 mg Q.d.	BASDAI, TNF⁃α, CRP, adverse events	38 ± 12	40 ± 10	6.21 ± 1.45	6.34 ± 1.19	–	–	24 weeks
Yan et al., 2021 (21)	China	48	25	Iguratimod 50mg Q.d + NSAIDs	NSAIDs + Placebo	BASDAI, BASFI, CRP, ESR, adverse events	31.38 ± 7.36	30.28 ± 5.94	4.69 ± 0.94	4.57 ± 0.57	3.41 ± 1.33	3.49 ± 1.23	24 weeks
Bai et al., 2021 (22)	China	43	43	Iguratimod 25mg B.i.d + Sulfasalazine 1g B.i.d + Celecoxib 200mg B.i.d	Sulfasalazine 1g B.i.d + Celecoxib 200mg B.i.d	BASDAI, VAS, CRP, ESR, adverse events	28.52 ± 9.43	27.87 ± 8.05	5.92 ± 0.96	5.88 ± 1.06	–	–	12 weeks
Li et al., 2021 (23)	China	30	30	Iguratimod 25mg B.i.d + Sulfasalazine 0.5 to 1g B.i.d + Thalidomide 50 to 200mg Qn	Sulfasalazine 0.5 to 1g B.i.d + Thalidomide 50 to 200mg Qn	BASDAI, TNF⁃α	31.24 ± 4.71	30.01 ± 4.68	37.47 ± 4.06	38.14 ± 4.37	–	–	24 weeks
Zhang et al., 2022 (24)	China	35	34	Iguratimod 25mg B.i.d + Celecoxib 0.2g Q.d. + Sulfasalazine 0.25 B.i.d	Celecoxib 0.2g Q.d. + Sulfasalazine 0.25 B.i.d	BASFI, CRP, ESR, SOD, CTX-I, TNF⁃α, adverse events	49~75	48~74	–	–	5.93 ± 1.41	5.89 ± 1.37	12 weeks

"-" indicates no data. "+" means plus.

### 3.2 Risk of bias of included studies

**Figure 2:**
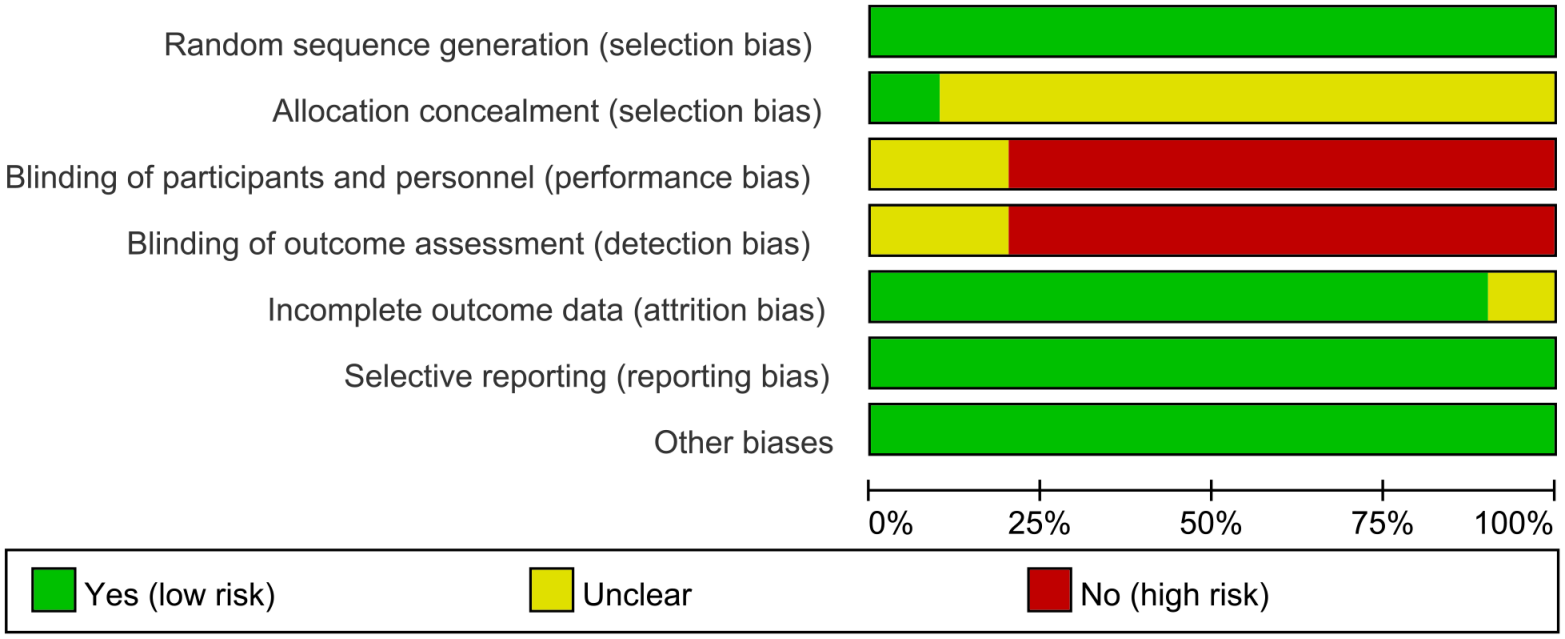
Risk of bias graph.

**Figure 3:**
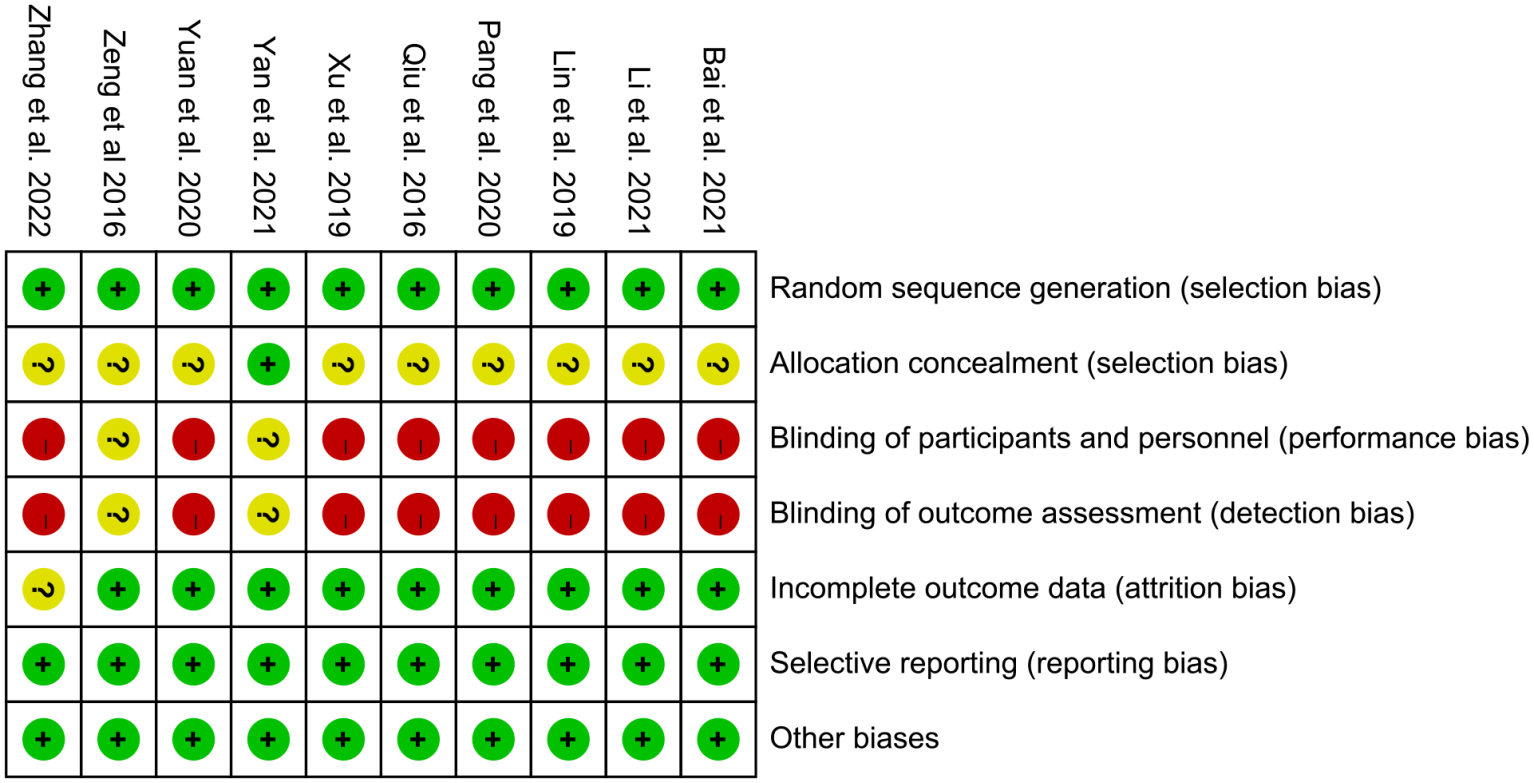
Risk of bias summary.

The summary and graph of risk of bias ware shown in **Figures 2**, **3**.

#### 3.2.1 Sequence generation and allocation concealment

The random sequences of all RCTs are generated by random number table method, so we evaluate them as low risk of bias. Meanwhile, only Yang et al. (21) describe an acceptable method of allocation concealment, while other RCTs did not describe an acceptable method of allocation concealment. Therefore, Yang et al. (21) were rated as having a low risk of bias, while others were rated as having an unclear risk of bias.

#### 3.2.2 Blinding

Zeng et al. (20) and Yang et al. (21) stated in the RCT that the blind method was used, but did not describe the specific implementation process of the blind method, so we thought its risk of bias is unclear. Other studies did not specify whether to use blinding, and their main outcome are subjective evaluation indicators (such as BASDAI, BASFI, VAS), which are easily affected by non-blinding, so we believe that their risk of bias is high.

#### 3.2.3 Incomplete outcome data and selective reporting

All RCTs do not have incomplete outcome data and selective reporting, so we evaluate them as low risk.

#### 3.2.4 Other potential bias

Other sources of bias were not observed in 8 RCTs; therefore, the risks of other bias of the RCTs were low.

### 33 Primary outcomes

#### 3.3.1 BASDAI

**Figure 4:**
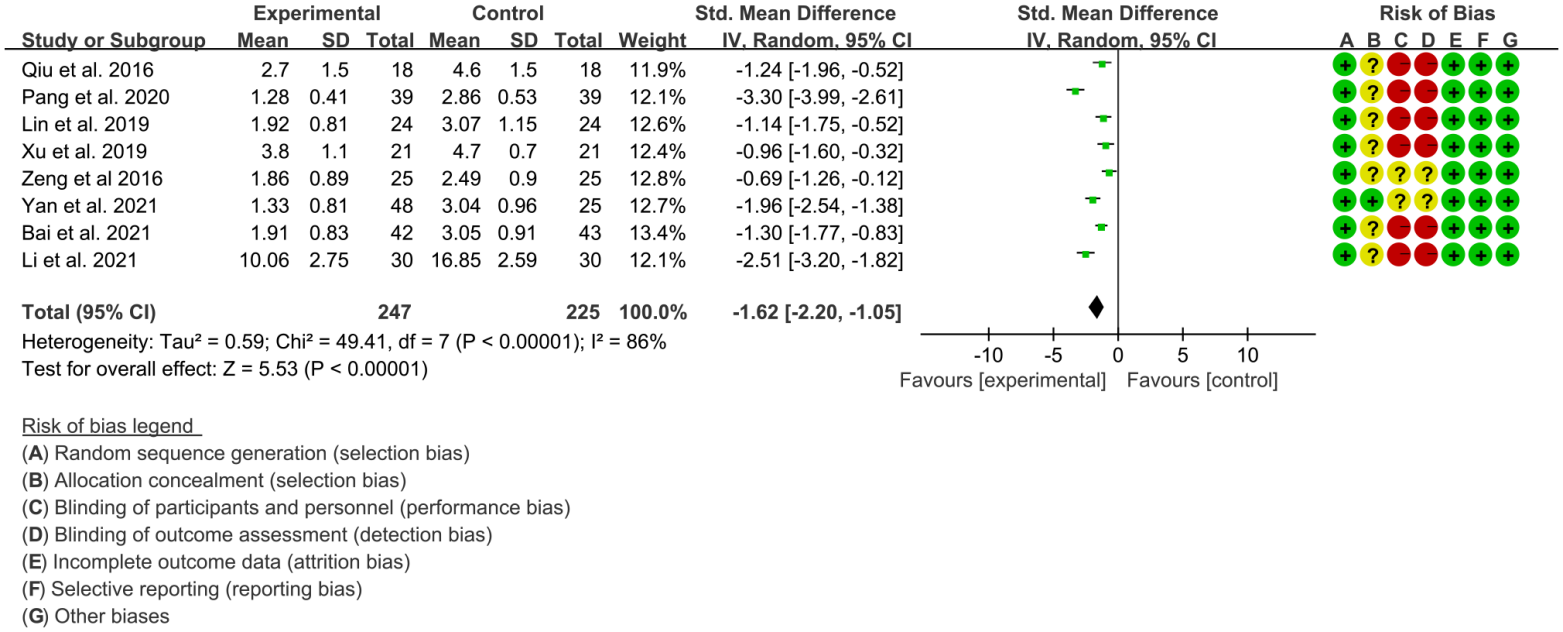
The results of BASDAI (CI, confidence interval; SD, standard deviation).

Eight RCTs (15, 17–23) utilized BASDAI to assess the improvement of AS, which include 247 patients in IGU group and 225 patients in control group. The heterogeneity test showed that P<0.00001, I2 = 86%, which suggest that the heterogeneity is high, and the random effects model was used for analysis. The meta-analysis results show that compared with the control group, the BASDAI in the IGU group was lower (SMD -1.62 [-2.20, -1.05], P<0.00001; random effect model) (**Figure 4**).

#### 3.32 BASFI

**Figure 5:**
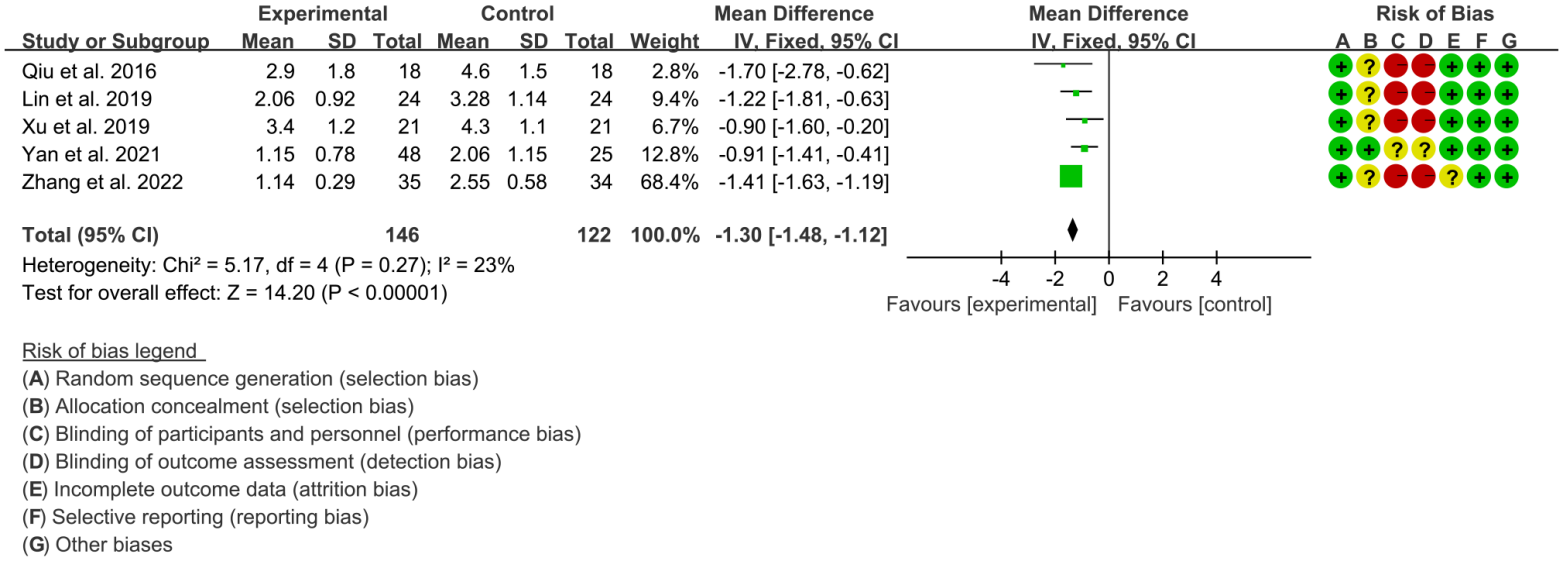
The results of BASFI (CI, confidence interval; SD, standard deviation).

Five RCTs (15, 18, 19, 21, 24) utilized BASFI to assess the improvement of AS, including 146 patients in IGU group and 122 patients in control group. The heterogeneity test showed that P=0.27, I2 = 23%, which suggest that the heterogeneity is low, and the fixed effects model was used for analysis. The meta-analysis results show that compared with the control group, the BASFI in the IGU group was lower (WMD -1.30 [-1.48, -1.12], P<0.00001; fixed effect model) (**Figure 5**).

#### 333 VAS

**Figure 6:**
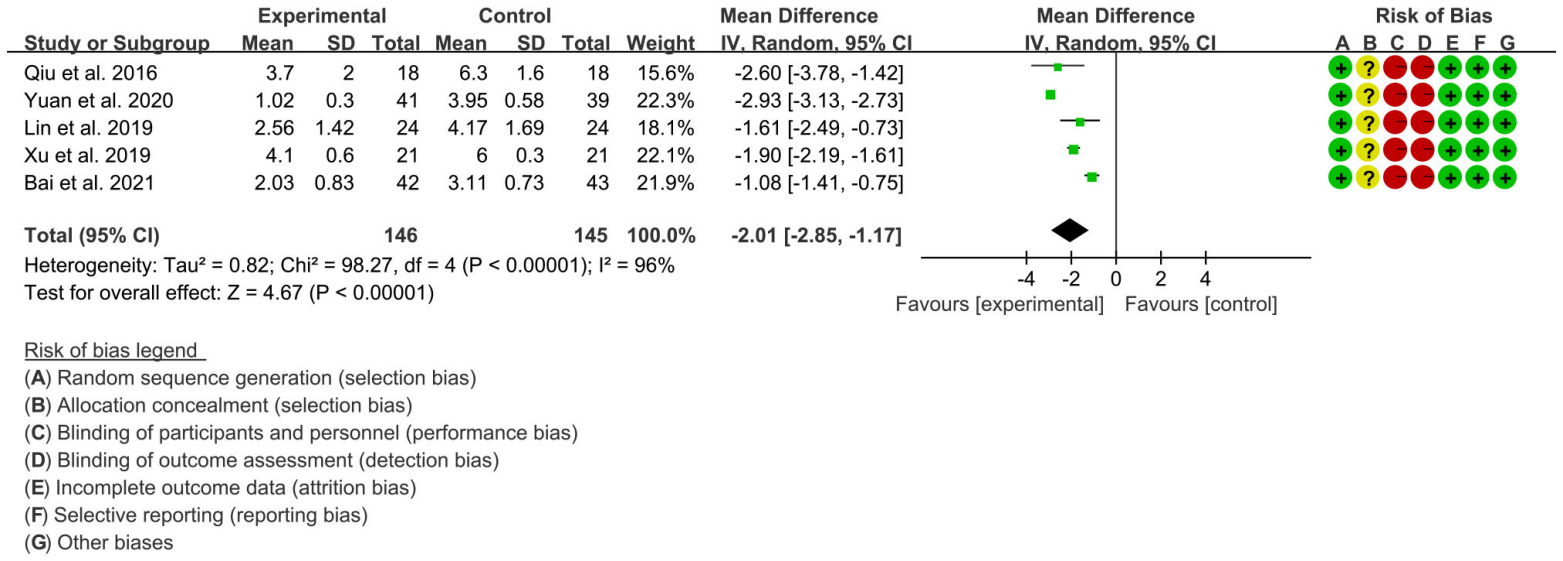
The results of VAS (CI, confidence interval; SD, standard deviation).

Four RCTs (15, 16, 18, 19, 22) utilized VAS to assess the improvement of AS, including 137 patients in IGU group and 135 patients in control group. The heterogeneity test showed that P<0.00001, I2 = 95%, which suggest that the heterogeneity is high, and the random effects model was used for analysis. The meta-analysis results show that compared with the control group, the VAS in the IGU group was lower (WMD -2.01 [-2.83, -1.19], P<0.00001; random effect model) (**Figure 6**).

### 3.4 Secondary outcomes

#### 3.4.1 The results of ESR

**Figure 7:**
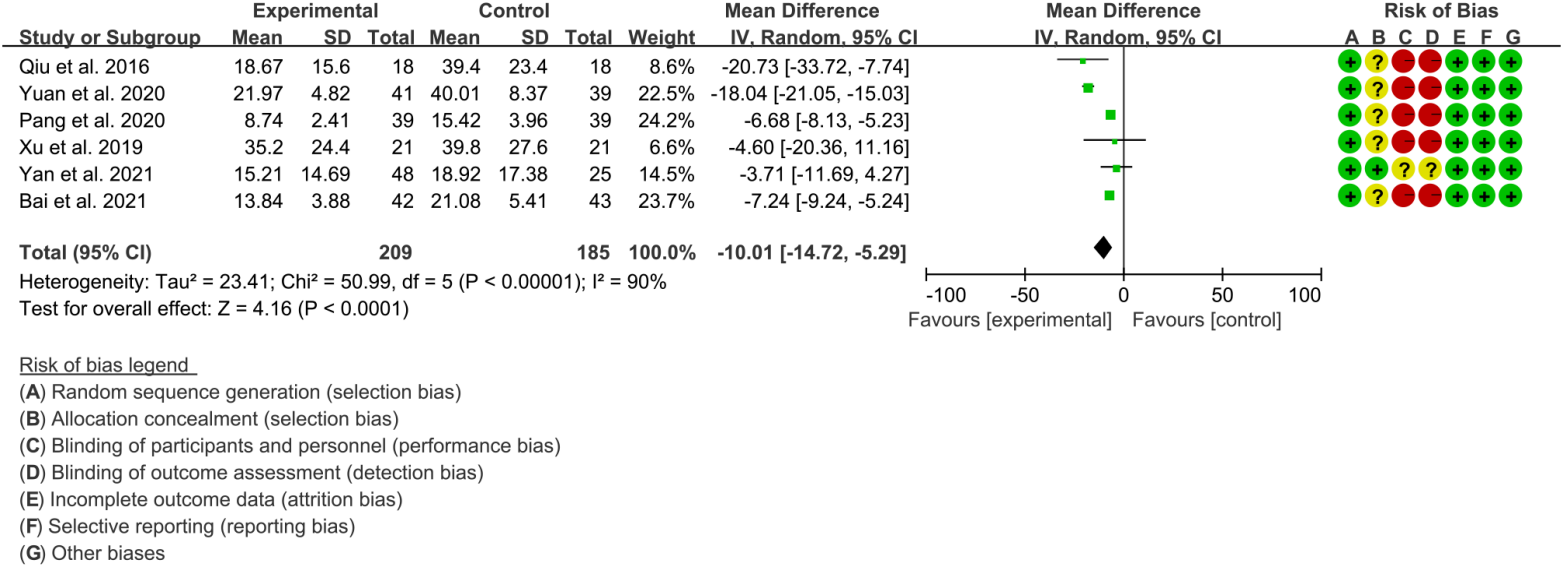
The results of ESR (CI, confidence interval; SD, standard deviation).

Six RCTs (15–17, 19, 21, 22) utilized ESR to assess the improvement of AS, which involves in 209 patients in IGU group and 185 patients in control group. The heterogeneity test showed that P<0.00001, I2 = 90%, which suggest that the heterogeneity is high, and the random effects model was used for analysis. The meta-analysis results show that compared with the control group, the ESR in the IGU group was lower (WMD -10.01 [-14.72, -5.29], P<0.0001; random effect model) (**Figure 7**).

#### 3.4.2 The results of CRP

**Figure 8:**
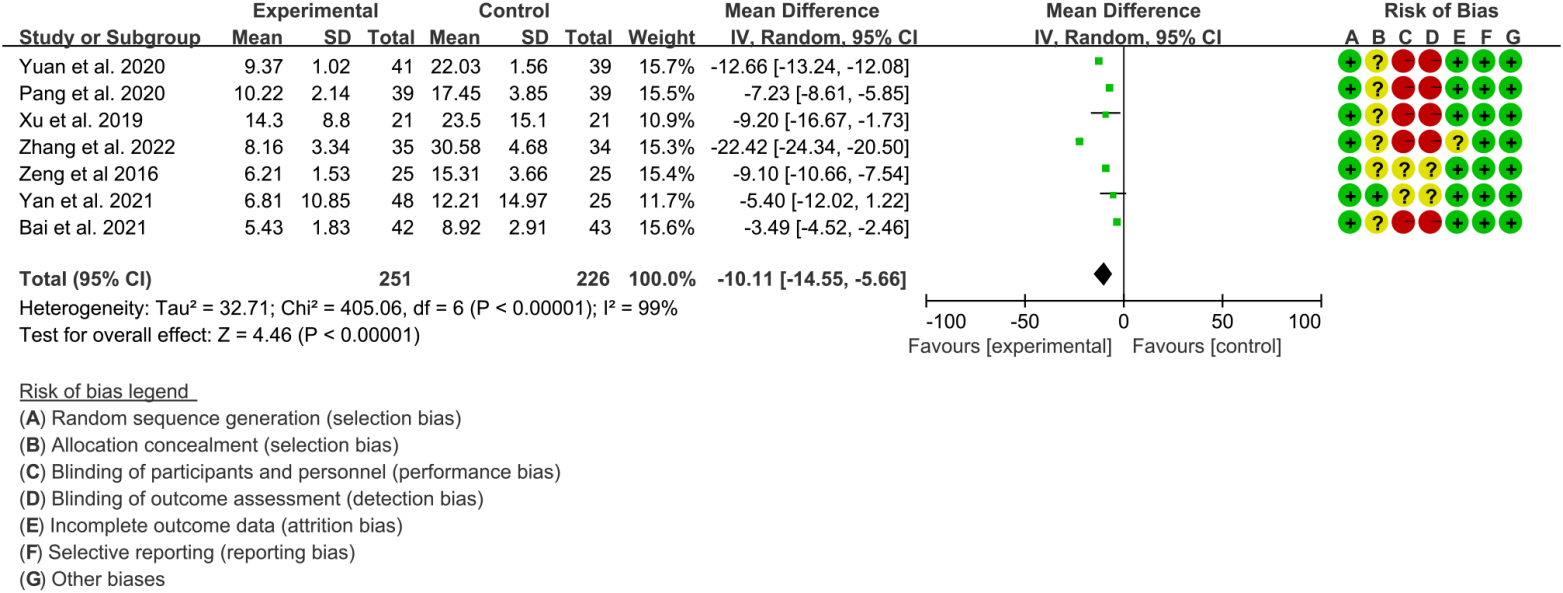
The results of CRP (CI, confidence interval; SD, standard deviation).

Seven RCTs (16, 17, 19–22, 24) utilized CRP to assess the improvement of AS, which involves in 251 patients in IGU group and 226 patients in control group. The heterogeneity test showed that P<0.00001, I2 = 99%, which suggest that the heterogeneity is high, and the random effects model was used for analysis. The meta-analysis results show that compared with the control group, the CRP in the IGU group was lower (WMD -10.11 [-14.55, -5.66], P<0.00001; random effect model) (**Figure 8**).

#### 3.4.3 The results of TNF-α

**Figure 9:**
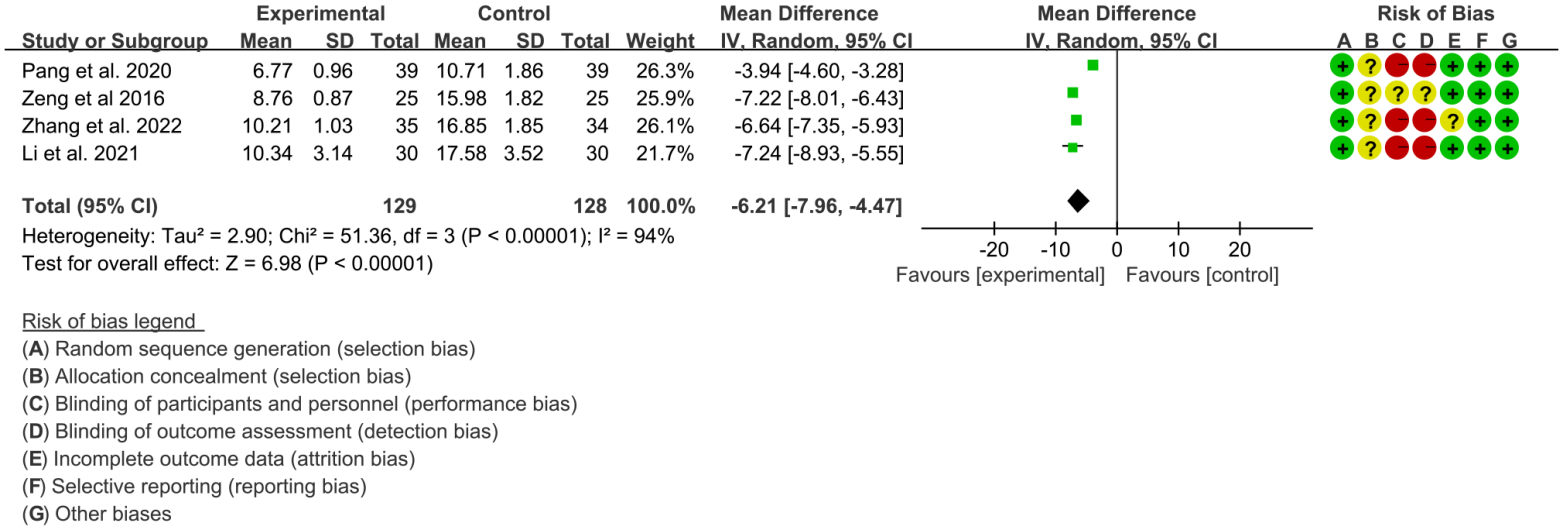
The results of TNF-α (CI, confidence interval; SD, standard deviation).

Four RCTs (18, 21, 23, 24) utilized TNF-α to assess the improvement of AS, which involves in 129 patients in IGU group and 128 patients in control group. The heterogeneity test showed that P<0.00001, I2 = 94%, which suggest that the heterogeneity is high, and the random effects model was used for analysis. The meta-analysis results show that compared with the control group, the TNF-α in the IGU group was lower (WMD -6.21 [-7.96, -4.47], P<0.00001; random effect model) (**Figure 9**).

#### 3.4.4 The results of SOD

**Figure 10:**
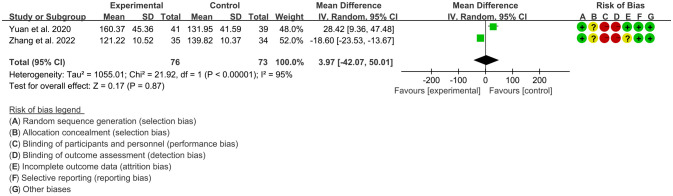
The results of SOD (CI, confidence interval; SD, standard deviation).

Two RCTs (16, 24) utilized SOD to assess the improvement of AS, which involves in 76 patients in IGU group and 73 patients in control group. The heterogeneity test showed that P<0.00001, I2 = 95%, which suggest that the heterogeneity is high, and the random effects model was used for analysis. The meta-analysis results show that there was no significant difference in SOD between the experimental group and the control group (WMD 3.97 [-42.07, 50.01], P=0.87; random effect model) (**Figure 10**).

#### 3.4.5 The results of CTX-I

**Figure 11:**
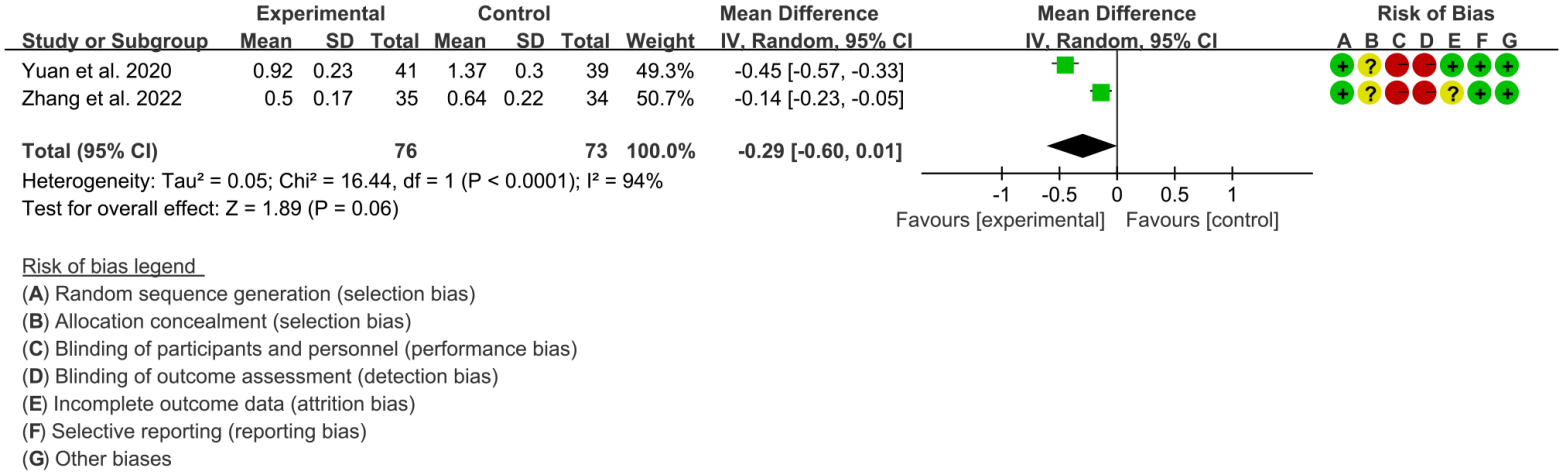
The results of CTX-I (CI, confidence interval; SD, standard deviation).

Two RCTs (16, 24) utilized CTX-I to assess the improvement of AS, which involves in 76 patients in IGU group and 73 patients in control group. The heterogeneity test showed that P<0.0001, I2 = 94%, which suggest that the heterogeneity is high, and the random effects model was used for analysis. The meta-analysis results show that there was no significant difference in CTX-I between the experimental group and the control group (WMD -0.29 [-0.60, 0.01], P=0.06; random effect model) (**Figure 11**).

#### 3.4.6 Other outcomes

Only Qiu et al. (16) reported back pain score, and they found that IGU can improve back pain score (P<0.05). Only Pang et al. (18) reported β⁃CTX and OPG levels, and they found that IGU can reduce β⁃CTX level and increase OPG level (P<0.05).

### 3.5 Adverse events

**Figure 12:**
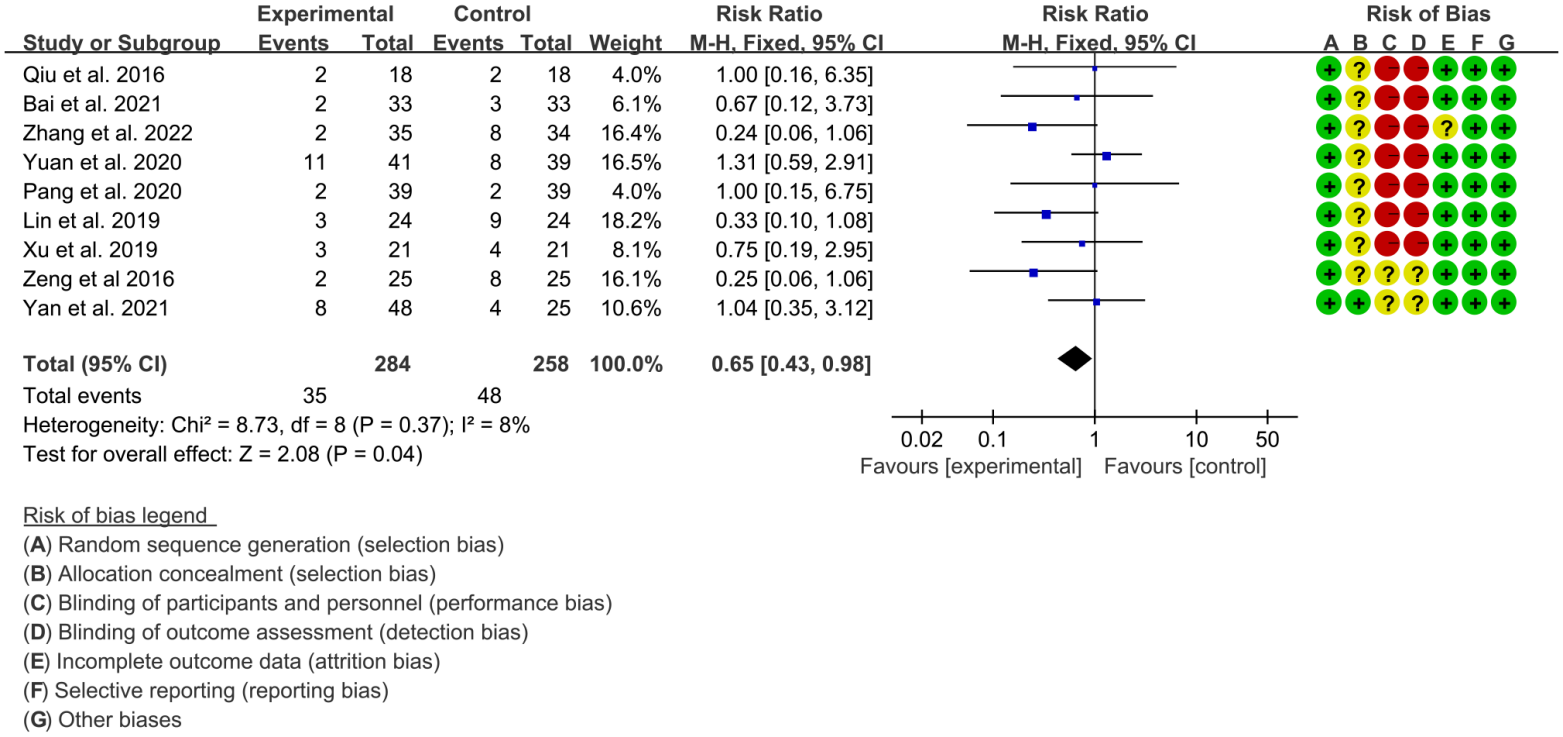
Adverse events (CI, confidence interval; SD, standard deviation).

Nine RCTs (15–22, 24) (284 patients in experimental group and 258 patients in control group) reported adverse events. The heterogeneity test P=0.37, I2 = 8%, indicating that the included studies are heterogeneous, and the fix effects model is used for analysis. The results of meta-analysis showed that incidence of adverse events in IGU group was lower (RR 0.65 [0.43, 0.98], P=0.04; fix effect model) (**Figure 12**).

### 3.6 Publication bias detection

**Figure 13:**
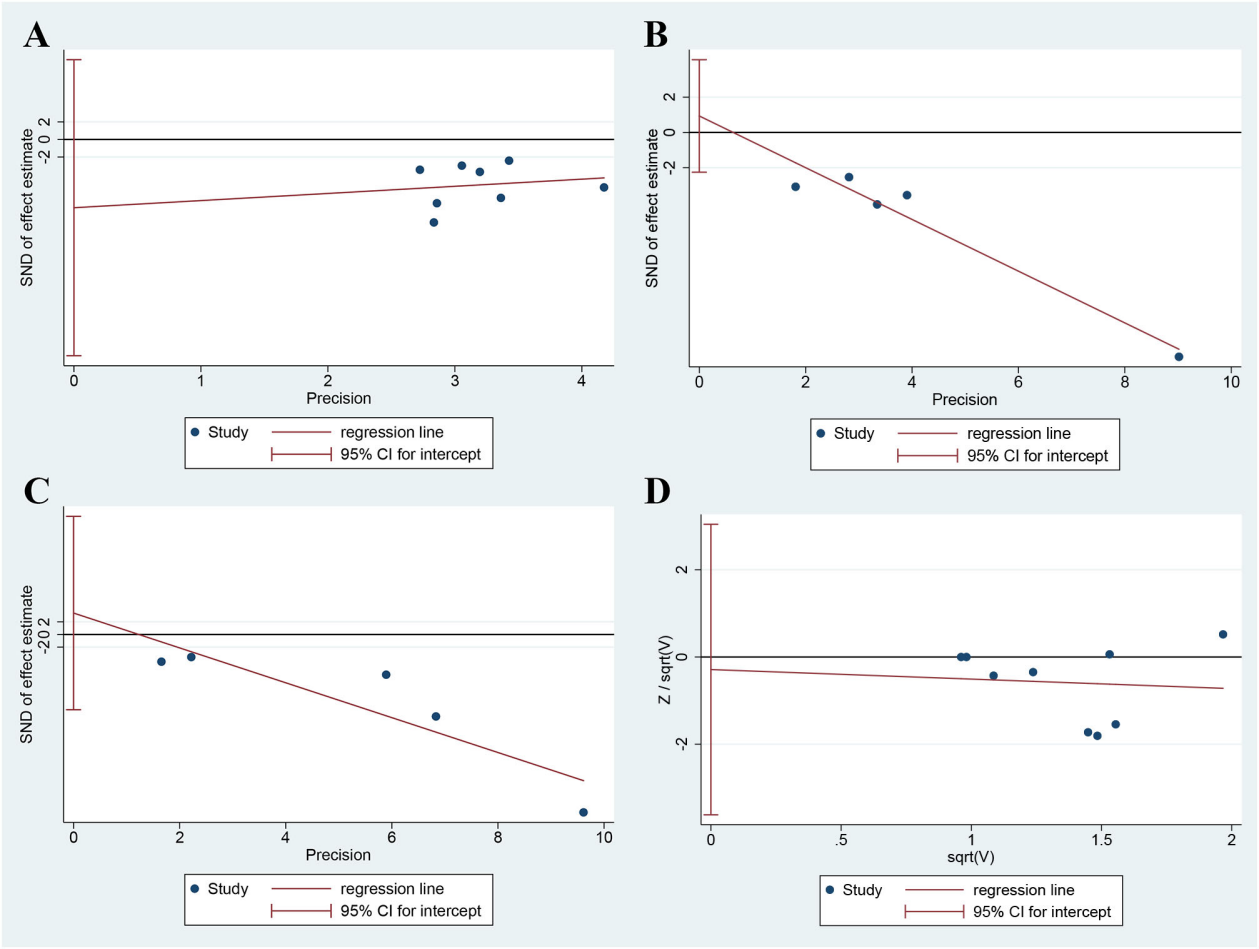
Publication Bias Detection **(A)** BASDAI; **(B)** BASFI; **(C)** VAS; **(D)** Adverse events.

The publication bias of the primary outcomes was detected by STATA 15.0. (1) BASDAI: The publication bias detection suggests that the possibility of publication bias was small (P=0.302) (**Figure 13A**). (2) BASFI: The publication bias detection suggests that the possibility of publication bias was small (P=0.420) (**Figure 13B**). (3) VAS: The publication bias detection suggests that the possibility of publication bias was small (P=0.531) (**Figure 13C**). (4) Adverse events: The publication bias detection suggests that the possibility of publication bias was small (P=0.844) (**Figure 13D**).

### 3.7 Subgroup analysis

The subgroup analysis was performed according to the duration (**Table 2**). The results of subgroup analysis showed that BASDAI, VAS, CRP, and TNF-α improved after 12 weeks of IGU treatment, and also improved after 24 weeks of treatment. However, for ESR, the addition of IGU treatment improved ESR at 12 weeks, while 24 weeks after the intervention showed no significant difference in ESR compared with the control group. For adverse events, the results showed that the 12-week intervention did not lead to an increase in the occurrence of adverse events, and the adverse events of long-term use (24 weeks) may be lower than that of the control group.

**Table 2:** Summary of findings for the main comparison.

Outcomes	Illustrative comparative risks* (95% CI)		Relative effect (95% CI)	No of Participants (studies)	Quality of the evidence (GRADE)	Comments
	Assumed risk	Corresponding risk				
	Control	Primary outcomes				
**Adverse events**	**Study population**		**RR 0.65** (0.43 to 0.98)	542 (9 studies)	⊕⊕⊕⊝ **moderate** ^1^	
	**186 per 1000**	**121 per 1000** (80 to 182)				
	**Moderate**					
	**191 per 1000**	**124 per 1000** (82 to 187)				
**BASDAI**		The mean basdai in the intervention groups was **1.62 standard deviations lower** (2.2 to 1.05 lower)		472 (8 studies)	⊕⊕⊝⊝ **low** ^1,2^	SMD -1.62 (-2.2 to -1.05)
**BASFI**		The mean basfi in the intervention groups was **1.3 lower** (1.48 to 1.12 lower)		268 (5 studies)	⊕⊕⊝⊝ **low** ^1,3^	
**VAS**		The mean vas in the intervention groups was **2.01 lower** (2.85 to 1.17 lower)		291 (5 studies)	⊕⊝⊝⊝ **very low** ^1,2,3^	

*The basis for the assumed risk (e.g. the median control group risk across studies) is provided in footnotes. The corresponding risk (and its 95% confidence interval) is based on the assumed risk in the comparison group and the relative effect of the intervention (and its 95% CI).

CI, Confidence interval; RR, Risk ratio;

GRADE Working Group grades of evidence.

High quality: Further research is very unlikely to change our confidence in the estimate of effect.

Moderate quality: Further research is likely to have an important impact on our confidence in the estimate of effect and may change the estimate.

Low quality: Further research is very likely to have an important impact on our confidence in the estimate of effect and is likely to change the estimate.

Very low quality: We are very uncertain about the estimate.

^1^ Downgraded one level due to serious risk of bias (random sequence generation, allocation concealment, blinding, incomplete outcomes) and most of the data comes from the RCTs with moderate risk of bias.

^2^ Downgraded one level due to the probably substantial heterogeneity.

^3^ Downgraded one level due to the total sample size fails to meet the optimal information size.

## 4 Discussion

This research included 10 RCTs with 622 participants. In addition to ClinicalTrials.gov, we also searched the Chinese Clinical Trial Registry and found that currently ongoing randomized controlled studies are: ChiCTR1800019227 and ChiCTR2000029112. The meta-analysis results showed that IGU can decrease the BASDAI score, BASFI score and VAS. IGU can also reduce inflammation levels (decreasing ESR, CRP and TNF-α). Most of the results are highly heterogeneous, especially VAS, ESR, CRP and TNF-α. It may be because both BASDAI and VAS are subjective measurement indicators, and the subjective feelings of patients with different RCTs are not uniform. ESR, CRP and TNF-α are individual biochemical indicators, and patients in different RCTs are different due to different conditions. All studies reported adverse reactions and no patient deaths were reported. Compared with the control group, the adverse events of the IGU group was lower. This shows that the addition of IGU will not cause additional adverse events to patients, and the occurrence of adverse events may be lower in IGU treatment over 24 weeks.

Current research shows that IGU, as a new type of anti-rheumatic drug, has good anti-inflammatory and immunosuppressive effects, and may be a potential drug for the treatment of AS in the future. The main clinical features of AS include inflammatory back pain caused by myositis and inflammation of other parts of the axial skeleton, peripheral arthritis, enteritis and anterior uveitis (30). In addition to inflammation, AS is also characterized by new bone formation in sacroiliac joints (SIJ) and the spine (31). Theories about the pathogenesis of AS include misfolding during the assembly of human leukocyte antigen (HLA)-B27, which leads to endoplasmic reticulum stress and unfolded protein response (UPR) (32). The activation of UPR gene leads to the release of TNF-α and IL-17, which is very important in the development of AS (33). The COX-2/PGE2 pathway is also important in the pathogenesis of AS (34). In addition, current evidence shows that MIF can promote inflammation and bone formation in AS (35). MIF also interacts with IL-17 and TNF-α pathways by up-regulating the expression and secretion of IL-17 and induces the production of TNF-α (35).

**Figure 14:**
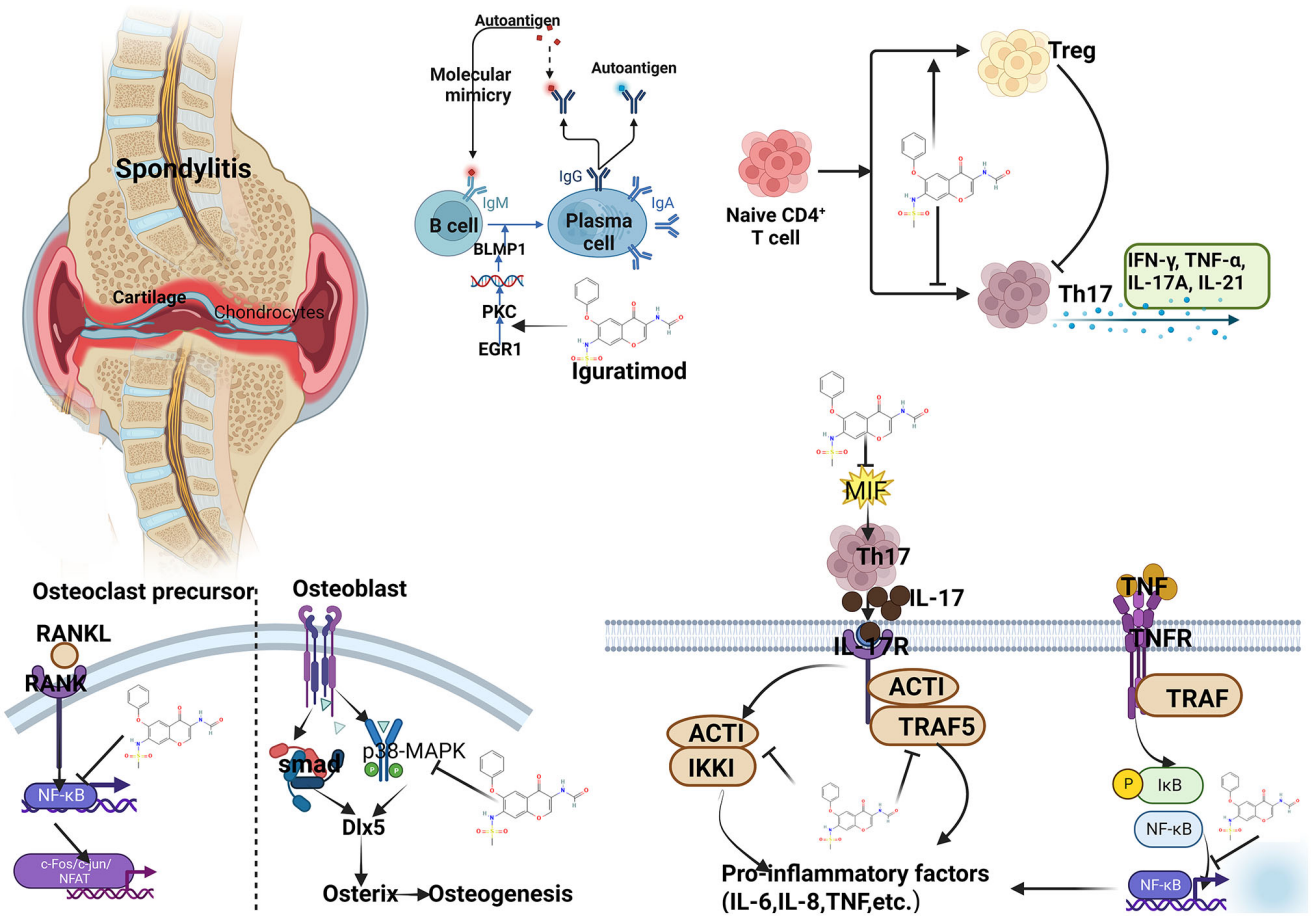
Summary of mechanism of IGU treating AS (PKC, protein kinase C; EGR1, early growth response 1; IFN-γ, interferon-γ; TNF-α, tumor necrosis factor-α; IL, interleukin; RANKL, NF-κB receptor activating factor ligand; MIF, Macrophage migration inhibitory factor; TRAF, tumor necrosis factor receptor-associated factor).

IGU plays an important role in suppressing immunity, inflammation, and maintaining bone balance. (1) In terms of inhibiting inflammatory factors and osteoclast intracellular signaling pathways: Bao et al. found in collagen-induced arthritis mice (CIA) that IGU can inhibit IL-17 expression while reducing TNF-α, IL-1β and IL-6 levels (36). Xu et al. confirmed that IGU can block the IL-17 pathway by targeting Act1, and IL-17 is an important cytokine involved in bone destruction in RA patients (37). The NF-κB pathway is an important intracellular conduction pathway in the process of osteoclast activation. Kohno et al. found that IGU can interfere with the translocation of NF-κB p65 into the nucleus and inhibit the activity of NF-κB (38). (2) In terms of inhibiting bone resorption: RANKL is an important signal to initiate osteoclast activation. Zhang et al. confirmed *in vitro* experiments that in mouse RAW264.7 cells, IGU can inhibit the number of osteoclasts induced by RANKL and reduce bone resorption pits (39). Guo et al. also found in bone marrow monocytes that IGU strongly inhibited RANKL-mediated osteoclastogenesis and bone resorption in a dose-dependent manner (40). IGU can also inhibit RANKL-induced osteoclast development and bone resorption in the PPARγ/c-Fos signaling pathway, and can also reduce the expression of downstream osteoclast marker genes (41). In addition, IGU not only inhibited the production of RANKL, but also significantly decreased the ratio of RANKL/OPG in serum and IL-1β-induced RA-FLSs (42). IGU inhibits the generation, differentiation, migration and bone resorption of osteoclasts induced by RANKL, and reduces the expression of nuclear activated T cell factor (NFAT) c1 and downstream osteoclast marker genes (43). These effects collectively show the effect of IGU attenuating bone erosion. Gan et al. found that IGU significantly inhibited RANKL-induced osteoclast differentiation, migration and bone resorption in RAW264.7 cells in a dose-dependent manner; the mechanism was related to the activation of MAPK and NF-κB pathways (44). It shows that IGU has a direct inhibitory effect on the formation and function of osteoclasts. In addition to osteoclasts, MMPs produced by FLSs also play an important role in cartilage destruction in spondylitis (45). Du et al. treated FLS with different doses of IGU *in vitro* and then stimulated them with TNF-α, IL-1β or IL-17A. MMP-3 was significantly inhibited by 5 μg/ml IGU, but MMP-1 was significantly inhibited at 50 μg/ml. Clinical trials found that after 24 weeks of IGU (25mg, 22 times a day) treatment, the levels of MMP-1 and MMP-3 were significantly reduced (46). All these suggest that IGU prevents MMP-1 and MMP-3 from protecting cartilage (43). (3) In terms of promoting bone formation: Kohji Kuriyama et al. found that IGU can promote the differentiation of mouse bone marrow stromal cells ST2 and embryonic osteoblast precursor cells MC3T3-E1 into osteoblasts *in vitro*, and can promote BMP-2 so as to induce bone formation *in vivo* (47). In addition, Osterix is a core transcription factor that regulates bone formation and plays a key role in the differentiation of osteoblasts (48), while IGU can increase the expression of Osterix and osteocalcin (41). Song et al. also found that IGU can increase the expression of Dlx5 and Osterix and regulate the p38 pathway to promote osteoblast differentiation and maturation in mesenchymal stem cells (49). (4) In the aspect of regulating immunity: IGU can regulate immune balance by regulating T cells and related cytokine levels. Studies have shown that IGU can significantly reduce the number of Th1, Th17, follicular helper T (Tfh) cells and related transcription factors and cytokine levels, increase the number of regulatory T cells (Treg) and related transcription factors and cytokine levels (50–52). IGU also reduced the apoptosis of peripheral blood mononuclear cells, the content of IFN-γ in CD3 + T cells and the level of IL-8 in peripheral blood (53). In addition, in regulating B cells, IGU can also inhibit PKC pathway and its downstream target EGR1, thereby inhibiting B cell terminal differentiation into mature plasma cells to reduce the production of autoantibodies (54). In summary, IGU can be controlled by multiple targets, and it can inhibit cartilage and bone destruction in the pathological process of AS, and has the basis of bone protection (see **Figure 14**).

To promote the conclusion, the GRADE tool was utilized to rate the quality of the evidence (55). According to the GRADE handbook (56), the evidence was judged to be moderate to very low (**Table 3**).

**Table 3:** Subgroup analysis results.

Outcomes	Subgroup	Overall effect			Heterogeneity test		Statistical method	Studies (N)	Sample size (N)	Figure
		Effect	95%CI	P	I^2^ (%)	P(Q)				
BASDAI	12 weeks	SMD=-2.28	[-4.25, -0.32]	0.023	95.47	<0.00001	Random	2	163	**Figure S1**
	24 weeks	SMD=-1.41	[-1.95, -0.87]	<0.00001	77.39	0.0005	Random	6	309
VAS	12 weeks	MD=-2.01	[-3.82, -0.20]	0.03	98.84	0.00001	Random	2	165	**Figure S2**
	24 weeks	MD=-1.91	[-2.17, -1.64]	0	0	0.42	Random	3	126
ESR	12 weeks	MD=-11.93	[-17.24, -6.62]	<0.0001	94.34	0.00001	Random	4	312	**Figure S3**
	24 weeks	MD=-9.24	[-20.13, 1.65]	0.096	59.78	0.083	Random	3	151
CRP	12 weeks	MD=-11.41	[-17.65, -5.17]	0.0003	99.25	0.00001	Random	4	312	**Figure S4**
	24 weeks	MD=-8.92	[-10.40, -7.43]	0	0	0.56	Random	3	165
TNF-α	12 weeks	MD=-5.29	[-7.93, -2.64]	0.00009	96.66	0.00001	Random	2	147	**Figure S5**
	24 weeks	MD=-7.22	[-7.94, -6.51]	0	0	0.98	Random	2	110
Adverse events	12 weeks	RR=0.78	[0.43, 1.41]	0.42	26.82	0.25	Fixed	4	293	**Figure S6**
	24 weeks	RR=0.55	[0.31, 0.97]	0.038	0	0.44	Fixed	5	249

The strengths of this review is that this we firstly conducted a systematic review and meta-analysis about IGU on AS. This study not only found that adding IGU to conventional therapy can improve AS, but also showed that it does not increase adverse reactions. However, the limitations is that most of the RCTs included this time did not use blinding, and did not hide the allocation of interventions, leading to a high risk of bias in the results. The number of RCTs included in this study is small, and the number of participants involved is not more than 1,000, which may affect the accuracy of the results. Moreover, most of the patients included in the study included this time are Chinese, which may affect the applicability of the results. Therefore, high-quality RCTs involving more countries and regions are needed in the future to revise or verify the results of this meta-analysis.

## 5 Conclusion

Through the systematic evaluation and meta-analysis of this study, it can be clarified that IGU as a new multi-targeted DMARD may have multiple benefits in the treatment of AS.

## Data availability statement

The original contributions presented in the study are included in the article/**Supplementary Material**. Further inquiries can be directed to the corresponding authors.

## Author contributions

LZ, YiD, KY, HC are responsible for the study concept and design. LZ, YiD, QH, ZL, KY, WH, YuD, JF, HC are responsible for the data collection, data analysis and interpretation. LZ and KY drafted the paper. HC supervised the study. All authors contributed to the article and approved the submitted version.
