# Supplementary material for: Efficacy and safety of Iguratimod in the treatment of Ankylosing Spondylitis: A systematic review and meta-analysis of randomized controlled trials
Source: Front Immunol. 2023 Mar 3;14:993860. doi: 10.3389/fimmu.2023.993860 (PMC10020631; doi:10.3389/fimmu.2023.993860)
Supplement: Supplementary file 1 [file Table_1.docx]

**Table S1.** Search Strategies for Pubmed and Embase

| **PubMed** | (Iguratimod OR Alamode OR T-614 OR C17H14N2O6S OR CAS 123663-49-0 OR IGU OR 3-Formylamino-7-methylsulfonylamino-6-phenoxy-4H-1-benzopyran-4-one)  AND  (Spondyloarthritis Ankylopoietica OR Ankylosing Spondylarthritis OR Ankylosing Spondylarthritides OR Spondylarthritides, Ankylosing OR Spondylarthritis, Ankylosing OR Ankylosing Spondylitis OR Spondylarthritis Ankylopoietica OR Bechterew Disease OR Bechterew's Disease OR Bechterews Disease OR Marie-Struempell Disease OR Marie Struempell Disease OR Rheumatoid Spondylitis OR Spondylitis, Rheumatoid OR Spondylitis Ankylopoietica OR Ankylosing Spondyloarthritis OR Ankylosing Spondyloarthritides OR Spondyloarthritides, Ankylosing OR Spondyloarthritis, Ankylosing)  AND  (random* controlled trial [pt] OR controlled clinical trial* [pt] OR randomized [tiab] OR placebo [tiab] OR drug therapy [sh] OR random* [tiab] OR trial* [tiab] OR group* [tiab])  NOT  (animals [mh] NOT humans [mh]) |
| --- | --- |
| **EMBASE** | 1 Iguratimod/  2 Alamode/  3 (T-614 or C17H14N2O6S or IGU).ti,ab.  4 1 or 2 or 3  5 Spondyloarthritis Ankylopoietica/  6 Ankylosing Spondylarthritis/  7 Ankylosing Spondylarthritides/  8 Ankylosing Spondylitis/  9 Spondylarthritis Ankylopoietica/  10 Bechterew Disease/  11 Bechterews Disease/  12 Marie-Struempell Disease/  13 Marie Struempell Disease/  14 Rheumatoid Spondylitis/  15 Spondylitis Ankylopoietica/  16 Ankylosing Spondyloarthritis/  17 Ankylosing Spondyloarthritides/  18 (AS).ti,ab.  19 5 or 6 or 7 or 8 or 9 or 10 or 11 or 12 or 13 or 14 or 15 or 16 or 17 or 18  20 4 and 20  21 randomized controlled trial/  22 single blind procedure/ or double blind procedure/  23 crossover procedure/  24 random*.tw.  25 (random or ((singl* or doubl*) adj (blind* or mask*)) or crossover or cross over or factorial* or latin square or assign* or allocat* or volunteer*).ti,ab.  26 21 or 22 or 23 or 24 or 25  27 (exp animals/ or nonhuman/) not human/  28 26 not 27  29 20 and 28 |
