# Supplementary figures and images for: Efficacy and safety of Iguratimod in the treatment of Ankylosing Spondylitis: A systematic review and meta-analysis of randomized controlled trials
Source: Front Immunol. 2023 Mar 3;14:993860. doi: 10.3389/fimmu.2023.993860 (PMC10020631; doi:10.3389/fimmu.2023.993860)

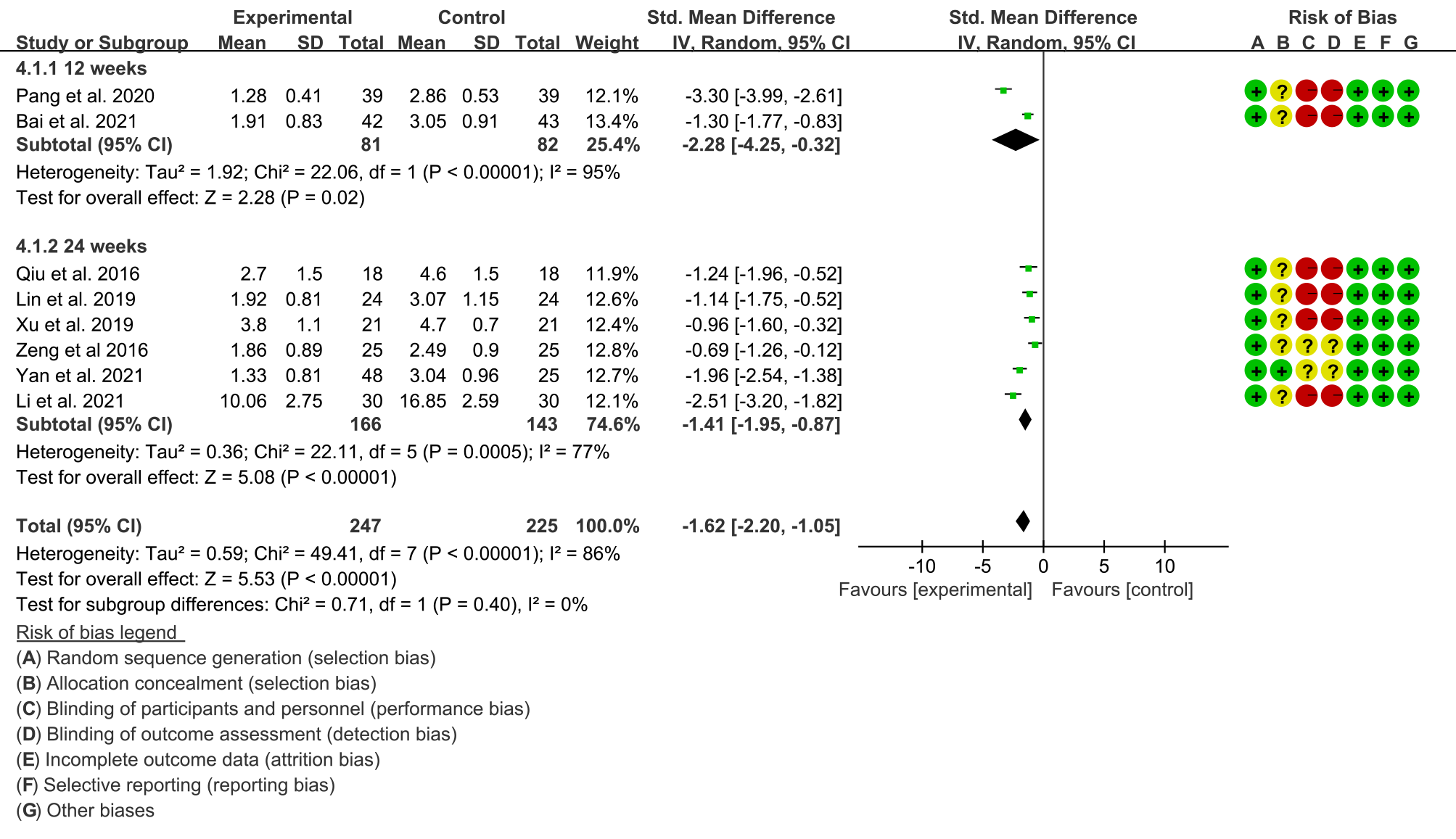

Supplement: Supplementary file 2 [file Image_1.tif]

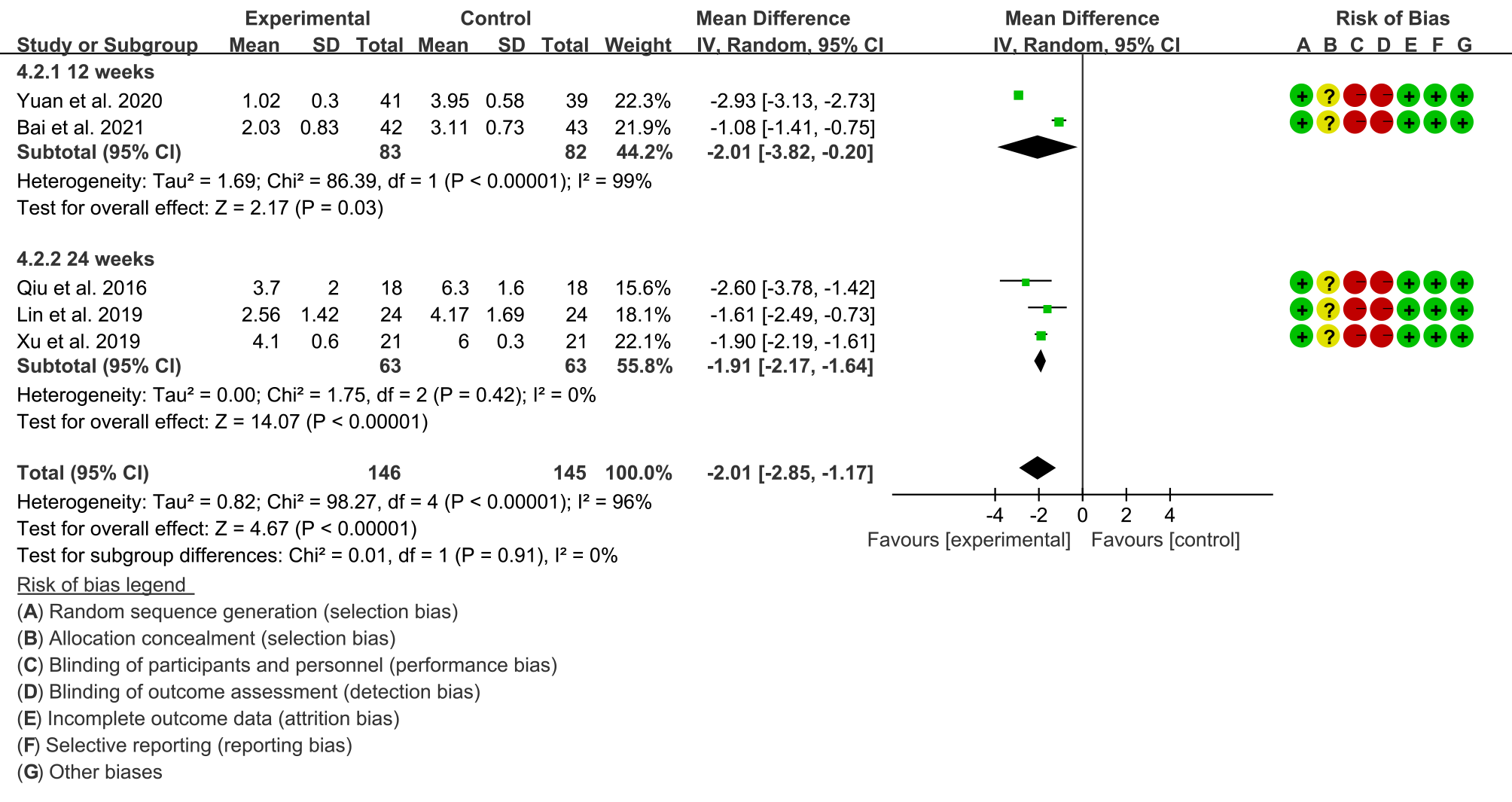

Supplement: Supplementary file 3 [file Image_2.tif]

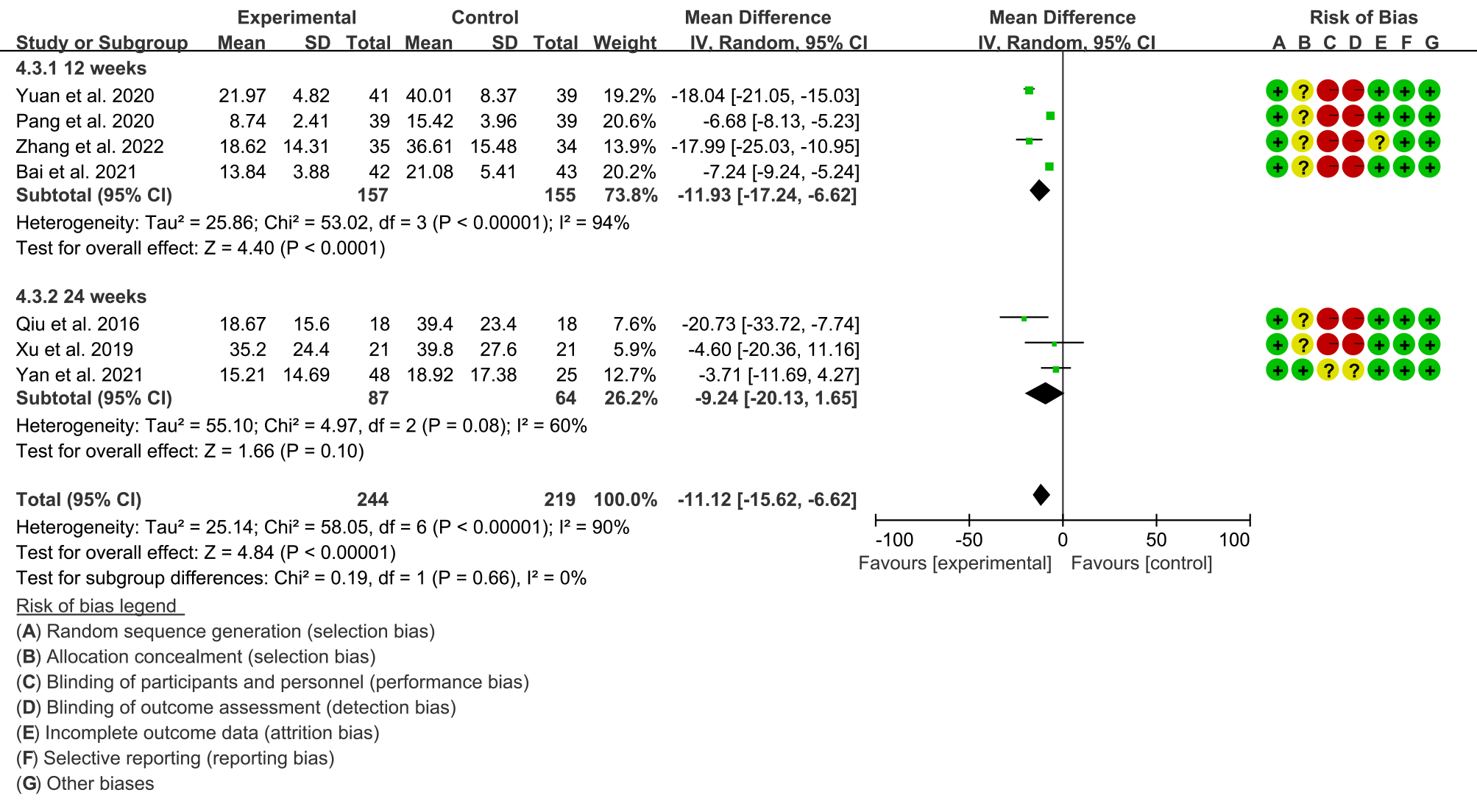

Supplement: Supplementary file 4 [file Image_3.tif]

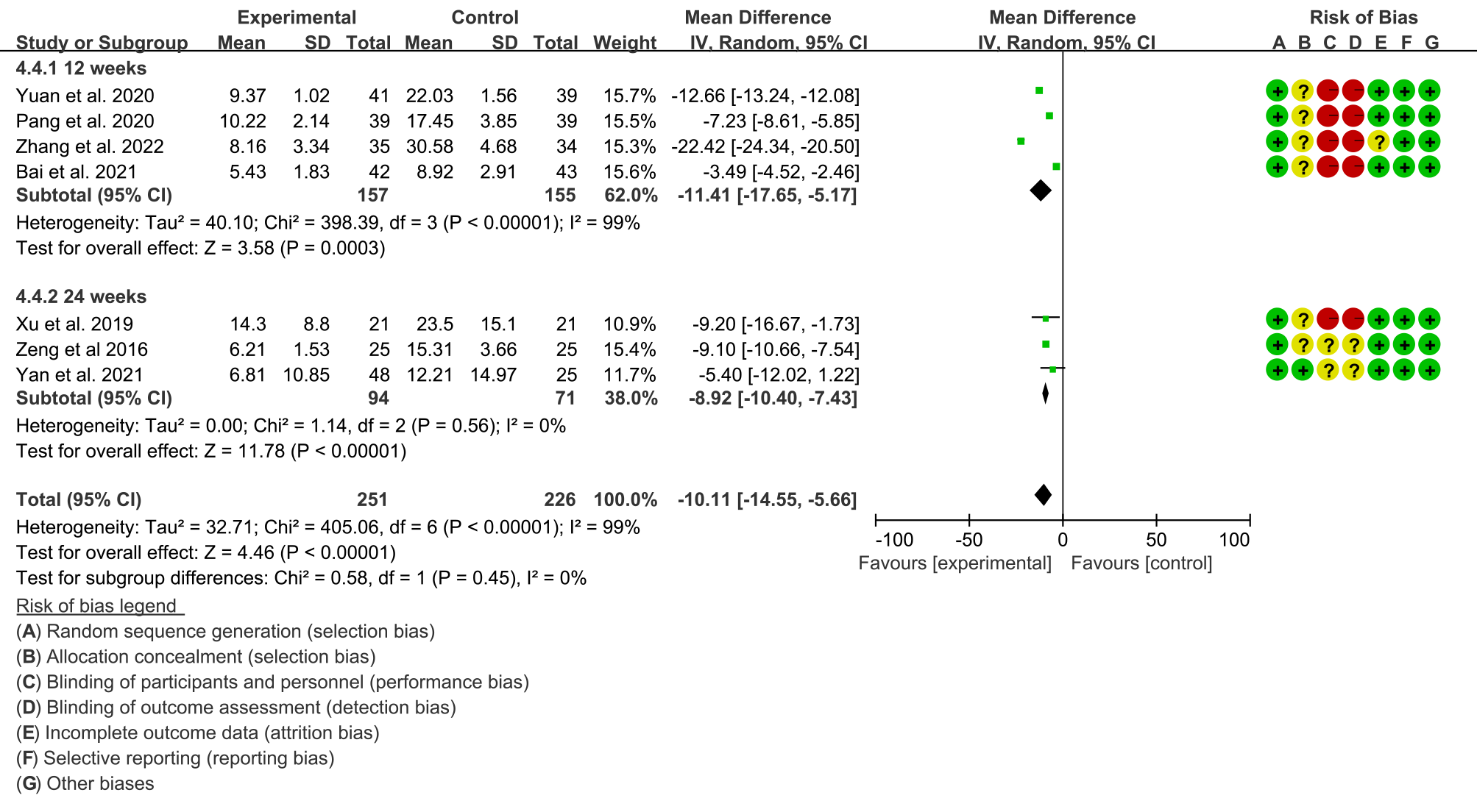

Supplement: Supplementary file 5 [file Image_4.tif]

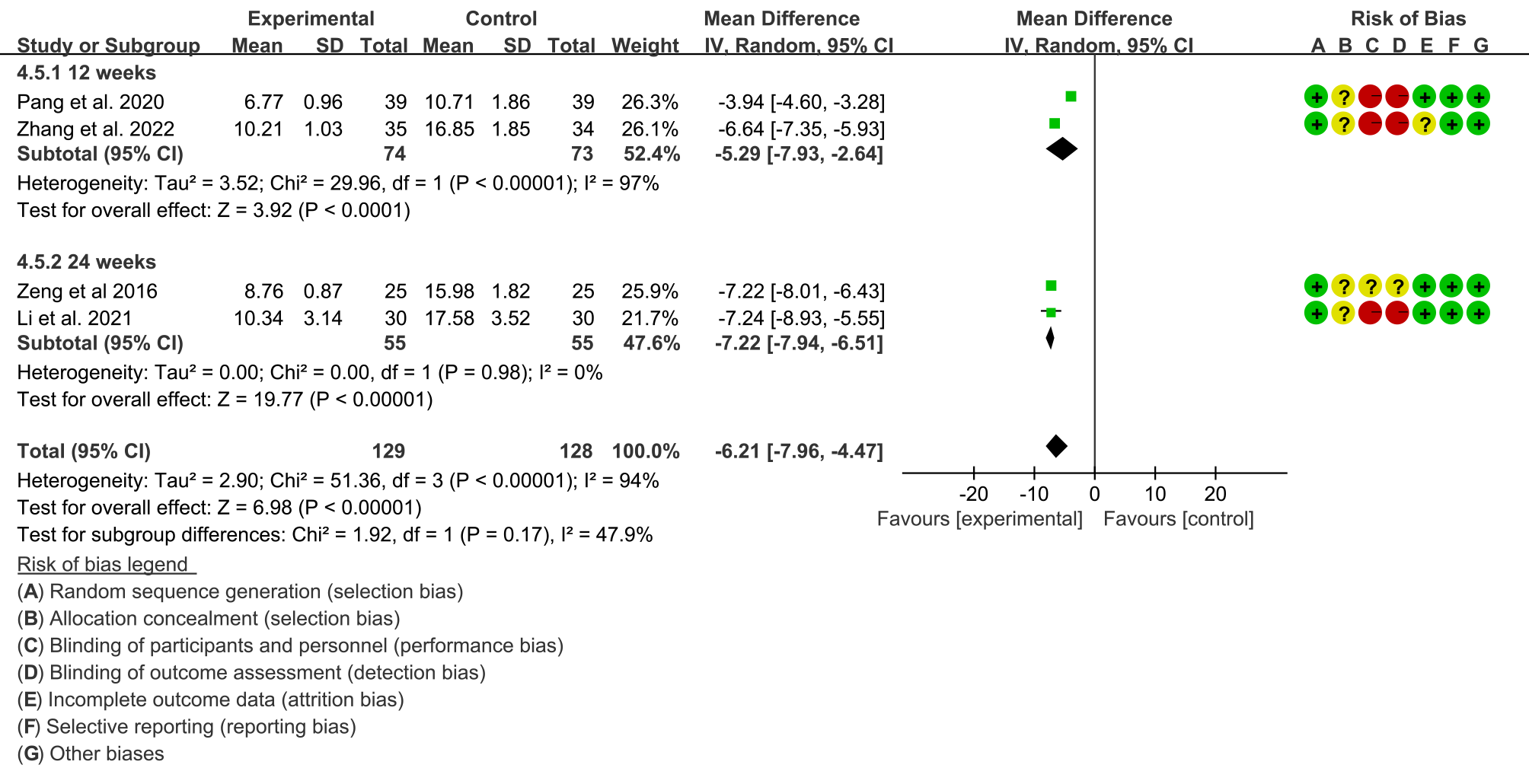

Supplement: Supplementary file 6 [file Image_5.tif]

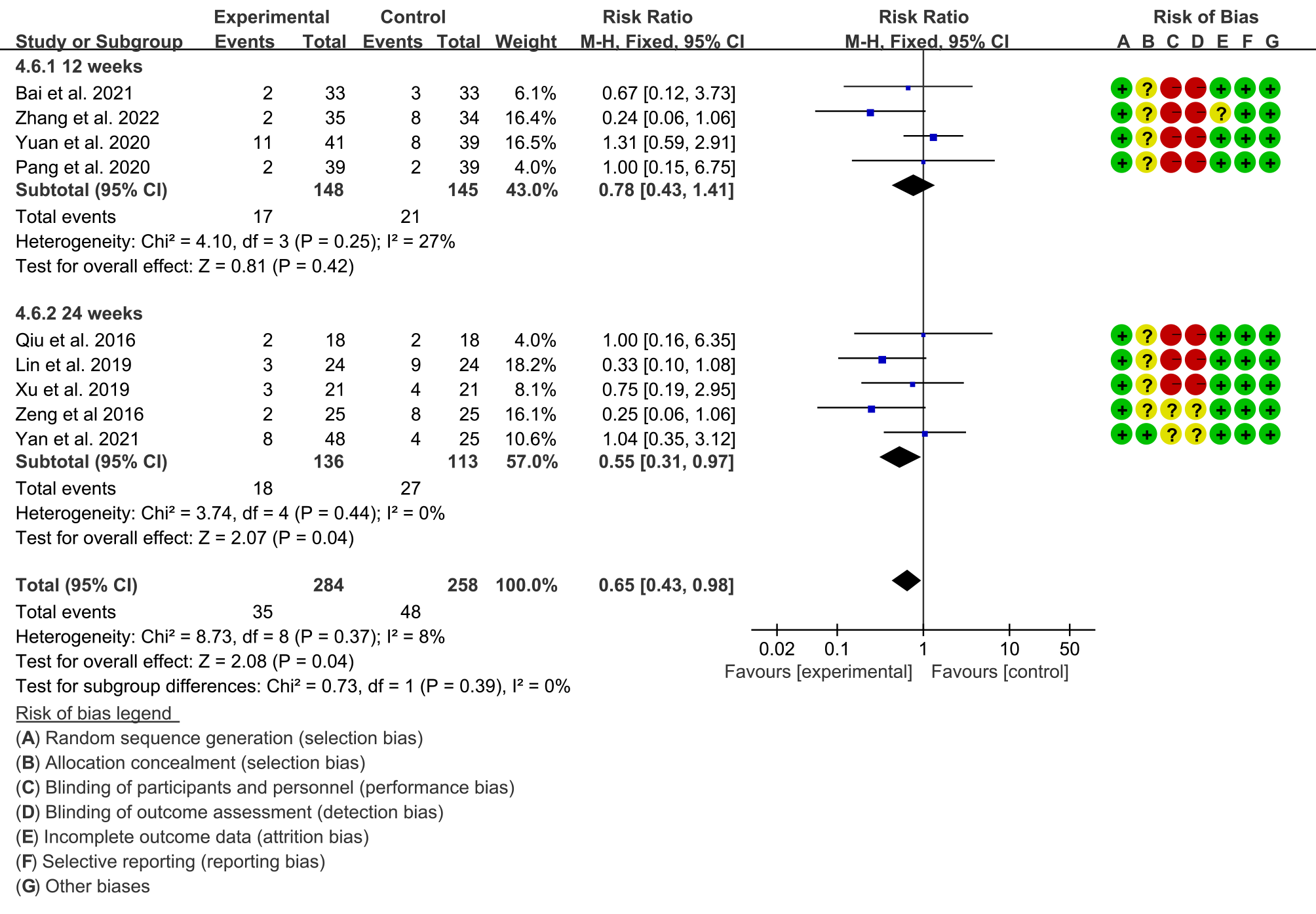

Supplement: Supplementary file 7 [file Image_6.tif]
